# Clofazimine for the treatment of tuberculosis

**DOI:** 10.3389/fphar.2023.1100488

**Published:** 2023-02-02

**Authors:** Jacob A. M. Stadler, Gary Maartens, Graeme Meintjes, Sean Wasserman

**Affiliations:** ^1^ Department of Medicine, University of Cape Town, Cape Town, South Africa; ^2^ Wellcome Centre for Infectious Diseases Research in Africa, Institute of Infectious Disease and Molecular Medicine, University of Cape Town, Cape Town, South Africa; ^3^ Department of Medicine, Division of Clinical Pharmacology, University of Cape Town, Cape Town, South Africa; ^4^ Division of Infectious Diseases and HIV Medicine, Department of Medicine, University of Cape Town, Cape Town, South Africa

**Keywords:** clofazimine, riminophenazines, B663, tuberculosis, drug-resistant tuberculosis (DR-TB), multidrug-resistant tuberculosis (MDR-TB)

## Abstract

Shorter (6–9 months), fully oral regimens containing new and repurposed drugs are now the first-choice option for the treatment of drug-resistant tuberculosis (DR-TB). Clofazimine, long used in the treatment of leprosy, is one such repurposed drug that has become a cornerstone of DR-TB treatment and ongoing trials are exploring novel, shorter clofazimine-containing regimens for drug-resistant as well as drug-susceptible tuberculosis. Clofazimine’s repurposing was informed by evidence of potent activity against DR-TB strains *in vitro* and in mice and a treatment-shortening effect in DR-TB patients as part of a multidrug regimen. Clofazimine entered clinical use in the 1950s without the rigorous safety and pharmacokinetic evaluation which is part of modern drug development and current dosing is not evidence-based. Recent studies have begun to characterize clofazimine’s exposure-response relationship for safety and efficacy in populations with TB. Despite being better tolerated than some other second-line TB drugs, the extent and impact of adverse effects including skin discolouration and cardiotoxicity are not well understood and together with emergent resistance, may undermine clofazimine use in DR-TB programmes. Furthermore, clofazimine’s precise mechanism of action is not well established, as is the genetic basis of clofazimine resistance. In this narrative review, we present an overview of the evidence base underpinning the use and limitations of clofazimine as an antituberculosis drug and discuss advances in the understanding of clofazimine pharmacokinetics, toxicity, and resistance. The unusual pharmacokinetic properties of clofazimine and how these relate to its putative mechanism of action, antituberculosis activity, dosing considerations and adverse effects are highlighted. Finally, we discuss the development of novel riminophenazine analogues as antituberculosis drugs.

## Introduction

Globally, nearly half a million new cases of multidrug- and rifampicin-resistant tuberculosis (MDR/RR-TB) are estimated to occur each year and this number is likely to increase due to the disruption of tuberculosis control efforts by the coronavirus disease (COVID) pandemic (World Health Organisation, 2021). For many years, treatment options for drug-resistant tuberculosis (DR-TB) were limited and required an extended treatment duration (≥18 months) using drugs with high toxicity and limited efficacy, including daily injections. The past decade has seen major developments in the DR-TB treatment landscape with considerable progress toward shorter, safer and more effective therapy through the use of new and repurposed drugs. The “BPaL/M” regimen (a combination containing bedaquiline, pretomanid and dose-optimised linezolid with or without moxifloxacin for 6- to 9-month duration) was recently recommended by the World Health Organisation (WHO) as the first-choice option for the treatment of MDR/RR-TB with or without additional resistance to fluoroquinolones (World Health Organisation, 2022a). This fully oral regimen is one of the most important milestones in tuberculosis treatment of the past decade, finally bringing the duration of treatment for DR-TB back down to that of standard therapy for drug-susceptible tuberculosis (DS-TB).

Clofazimine, a repurposed anti-leprosy drug, is recommended as a key drug in shorter as well as longer DR-TB regimens (World Health Organisation, 2020). Though the “BPaL/M” regimen excludes clofazimine, it remains an important drug option for individualised DR-TB therapy and is being evaluated in ongoing trials (summarised in Table 1) as a component of novel, shorter regimens for both DR- and DS-TB. This review presents an overview of the evidence underpinning the use and limitations of clofazimine as an antituberculosis drug. The unusual pharmacokinetic properties of clofazimine and how these relate to its putative mechanism of action, antituberculosis activity, dosing considerations and adverse effects are highlighted. Finally, we discuss the development of novel riminophenazine analogues as antituberculosis drugs.

**TABLE 1:** Clinical studies reporting efficacy and safety outcomes with regimens containing clofazimine (without* bedaquiline and linezolid) in adult patients with drug-resistant tuberculosis.

Study	Country	Study type	Regimen	Clofazimine dose	Regimen duration	Target population	HIV positive	Sample size	Number receiving clofazimine	Treatment success	Treatment failed	Died	Lost to follow-up	Sputum culture conversion rate	Incidence of skin discolouration
Mitnick, 2008 (Mitnick et al., 2008)	Peru	Retrospective, observational cohort	Individualized background regimen consisting of ≥ 5 of the following drugs: EMB, PZA, AMK, STR, KNM, CPM, CFX, OFX, LFX, SFX, CYS, ETO, PAS, CAC, CLM, CFZ, RFB	200–300 mg/d	Variable - individualized based on sputum culture results (median = 24.9 months)	MDR-TB and XDR-TB	1.4% (9/651)	651	447	MDR-TB: 66.3% (400/603) XDR-TB: 60.4% (29/48)	MDR-TB: 2.1% (13/603) XDR-TB: 10.4% (5/48)	MDR-TB: 20.4 (123/603) XDR-TB: 22.9% (11/48)	MDR-TB: 10.3% (62/603) XDR-TB: 6.2% (3/48)	Median time: MDR-TB: 61 days XDR-TB: 90 days	N/R
Van Deun, 2010 (Van Deun et al., 2010)	Bangladesh	Prospective, observational cohort	Six different standardized regimens used sequentially in consecutive cohorts over the study period. Regimens consisted of different combinations of the following drugs: GFX, OFX, KMC, CFZ, EMB, INH, PTO, PZA. (The most effective regimen consisted of KMC, GFX, CFZ, EMB, INH, PZA, PTO with CFZ + GFX given throughout)	100 mg/d (≥33 kg) 50 mg/d (<33 kg)	9–15 months (Most effective regimen = 9 months)	MDR-TB	Not tested (Reported as “virtually absent” in local population)	427	427 (Intensive phase only: 184; intensive and continuation phase: 243)	Overall: 78.3% (334/427) Most effective regimen: 87.9% (181/206)	Overall: 4.0% (17/427) Most effective regimen: 0.5% (1/206)	Overall: 7.7% (33/427) Most effective regimen: 5.3% (11/206)	Overall: 9.6% (41/427) Most effective regimen: 5.8% (12/206)	N/R	0%
Xu, 2012 (Xu et al., 2012b)	China	Retrospective, observational cohort	Individualized background regimen consisting of ≥ 4 of the following drugs including CFZ: AMK, CAC, AZM, CLM, CPM, EMB, GFX, LFX, MFX, OFX, PNH, PAS, PTO, PZA, INH, RPT, RFB, STR, LZD	100 mg/d	Variable - individualized based on sputum culture results	MDR-TB and XDR-TB	0%	39	39	38% (15/39)	23% (9/39)	0%	10% (4/39)	Median time: 12 weeks Proportion: 56.4% (22/39) *Time frame not specified	79.5% (31/39) 16/31 required CFZ dose adjustment or interrupted due to skin discolouration; 1 patient developed depression reportedly due to skin discolouration
Aung, 2014 (Aung et al., 2014)	Bangladesh	Prospective, observational cohort	Standardized regimen: GFX, CFZ, EMB, PZA, KNM, PTO, INH	100 mg/d (≥33 kg) 50 mg/d (<33 kg)	9–12 months	MDR-TB	Not tested (Reported as “virtually absent” in local population)	515	515	84.5% (435/515)	1.4% (7/515)	5.6% (29/515)	7.8% (40/515)	Proportion: 93% at 2 months	N/R
Padayatchi, 2014 (Padayatchi et al., 2014)	South Africa	Retrospective, observational cohort	Individualized background regimen consisting of a combination of the following drugs ± CFZ: PZA, CPM, ETO, MFX, OFX, PAS, TRD, EMB, INH, CAC, CLM, RIF	200–300 mg/d	Variable - individualized based on sputum culture results. (Follow-up limited to 12 months after treatment initiation.)	XDR-TB	CFZ group: 88.0% Control group: 82.9%	85	50	N/R	N/R	CFZ group: 36.0 Control group: 54.3	CFZ group: 58% (29/50) Control group: N/R	Median time: CFZ group: 16.4 weeks Control group: 11.9 weeks Culture conversion rate through 6 months favoured CFZ group: adjusted hazard ratio = 2.54, 95% CI: 0.99–6.52 Proportion at 12 months: CFZ group: 40% (20/50) Control group: 28.6% (10/35)	CFZ discontinued in 1 patient due to skin discolouration. 'Skin reaction' reported in 14% of those with adverse event data available (*n* = 42). Unclear if this refers to discolouration or other skin reactions
Piubello, 2014 (Piubello et al., 2014)	Niger	Prospective, observational cohort	Standardized regimen: GFX, CFZ, EMB, PZA, KNM, PTO, INH	100 mg/d (≥33 kg) 50 mg/d (<33 kg)	12–14 months	MDR-TB	1.7%	65	65	89.2% (58/65)	0%	9.2% (6/65)	1.6% (1/65)	Proportion: 93.8% at 4 months 100% at 6 months	3.1%
Kuaban, 2015 (Kuaban et al., 2015)	Cameroon	Prospective, observational cohort	Standardized regimen: GFX, CFZ, EMB, PZA, KNM, PTO, INH	100 mg/d	12–14 months	MDR-TB	20%	150	150	89.3% (134/150)	0.6% (1/150)	6.67% (10/150)	3.33% (5/150)	Proportion: 99.2% at 3 months	N/R
Tang, 2015 (Tang et al., 2015)	China	Randomized controlled trial	Individualized background regimen consisting of ≥ 5 of the following drugs ± CFZ: PTO, PZA, MFX/LFX/GFX, PAS, CPM/AMK, EMB, CLM	100 mg/d	21 months	MDR-TB	0%	105	53	CFZ arm: 73.6% (39/53) Control arm: 53.8% (28/52)	CFZ arm: 11.3% (6/53) Control arm: 28.8% (15/52)	CFZ arm: 7.5% (4/53) Control arm: 7.7% (4/52)	CFZ arm: 7.5% (4/52) Control arm: 9.6% (5/52)	Point estimates of median time not reported, but Kaplan-Meier analysis favoured CFZ arm (Log-rank *p* = 0.042)	94.3%
Dalcolmo, 2017 (Dalcolmo et al., 2017)	Brazil	Retrospective, observational cohort	CFZ group (2000–2006): AMK, OFX, TRD, EMB, STR, CFZ Control group (2006-2010): AMK, LFX, TRD, EMB, STR, PZA	100 mg/d (≥45 kg) 50 mg/d (<45 kg)	18 months	MDR-TB, pre-XDR-TB, XDR-TB	CFZ group: 5.5% Control group: 7.0%	2,542	1,446	CFZ arm: 60.9% (880/1,446) Control arm: 64.6% (708/1,096)	CFZ arm: 5.4% (78/1,446) Control arm: 8.7% (95/1,096)	CFZ arm: 23.7% (343/1,446) Control arm: 13.0% (142/1,096)	CFZ arm: 10.0% (144/1,446) Control arm: 13.8% (151/1,096)	N/R	50.2%
Trebucq, 2018 (Trebucq et al., 2018)	Multi-country (West and Central Africa)	Prospective, observational cohort	KNM, MFX, EMB, PZA, PTO, INH, CFZ	N/R	9–11 months	MDR-TB	19.9%	1,006	1,006	81.6% (821/1,006)	5.9% (59/1,006)	7.8% (78/1,006)	4.8% (48/1,006)	N/R	N/R
Wang, 2018 (Wang et al., 2018)	China	Randomized controlled trial	Individualized background regimen consisting of the following drugs ± CFZ: CPM/AMK, MFX/LFX, PZA, EMB, PAS, PTO	100 mg/d	36 months	XDR-TB	0%	49	22	CFZ arm: 36.4% (8/22) Control arm: 44.4% (12/27)	CFZ arm: 31.8% (7/22) Control arm:29.6% (8/27)	CFZ arm: 9.1% (2/22) Control arm: 11.1% (3/27)	CFZ arm: 22.7% (5/22) Control arm: 14.8% (4/27)	Median time: CFZ arm: 19.7 months Control arm: 20.3 months	22.7%
Duan, 2019 (Duan et al., 2019)	China	Randomized controlled trial	Individualized background regimen consisting of the following drugs ± CFZ: CPM/AMK, LFX, PZA, EMB, PAS, PTO, CAC	100 mg/d	24 months	MDR-TB	0%	140	66	CFZ arm: 65.1% (32/66) Control arm: 47.3% (35/74)	CFZ arm: 13.6% (9/66) Control arm: 32.4% (24/74)	CFZ arm: 6.1% (4/66) Control arm: 2.7% (2/74)	CFZ arm: 15.2% (10/66) Control arm: 15.6% (13/74)	Point estimates median time not reported, but Kaplan-Meier analysis favoured CFZ arm (Log-rank *p* = 0.031)	12.1%
Nunn, 2019 (Nunn et al., 2019)	Multi-country (Africa and Asia)	Randomized controlled trial	Short regimen (experimental): MFX, CFZ, EMB, PZA, KNM, INH, PTO Long regimen (control): Individualised as per local standard of care based on WHO guidelines. CFZ was part of standard of care in South Africa only as an optional drug	100 mg/d (≥33 kg) 50 mg/d (<33 kg)	Experimental arm (short regimen): 9–11 months Control arm (long regimen): 18–20 months	MDR-TB	32.6%	Efficacy mITT population: 369	Short regimen: 245 Long regimen: N/R	Short regimen: 78.8% (193/245) Long regimen: 79.8% (99/124)	Short regimen: 10.6% (26/245) Long regimen: 5.6% (7/124)	Short regimen: 3.7% (9/245) Long regimen: 4.0% (5/124)	Short regimen: 0.4% (1/245) Long regimen: 2.4% (3/124)	Point estimates median time not reported, but survival analysis found no difference between regimens, hazard ratio (95% CI): 1.16 (0.93–1.45)	No reports of skin discolouration. Unclear if this was due to non-occurrence or because it was not viewed as an adverse event
Du, 2020 (Du et al., 2020)	China	Randomized controlled trial	Experimental: CPM, LFX, CFZ, PTO, PZA Control: CPM, EMB, CYS, LFX, PTO, PZA	N/R	Experimental arm: 12 months Control arm: 18 months	MDR-TB	0%	135	67	Experimental arm: 68.7% (46/67) Control arm: 64.7% (44/68)	Experimental arm: 10.4% (7/67) Control arm: 14.7 (10/68)	Experimental arm: 3% (2/67) Control arm: 1.5% (1/68)	Experimental arm: 17.9% (12/67) Control arm: 19.1% (13/68)	Point estimates median time not reported, but Kaplan-Meier analysis did not find a significant difference between arms. Proportion at 3 months: Experimental arm: 68.7% Control arm: 55.9%	10.4%
Misra, 2020 (Misra et al., 2020)	South Africa	Prospective, observational cohort	Unspecified individualized background regimens including CFZ. Some received regimens containing BDQ or LZD.	100–300 mg/d	N/R	MDR-TB, pre-XDR-TB, XDR-TB	77.2%	600	<200 mg/d: 169 ≥ 200 mg/d: 431	Overall: 46.5% (279/600) <200 mg/d: 42.6% (72/169) ≥200 mg/d: 48% (207/431)	N/R	N/R	N/R	N/R	N/R

Abbreviations: AMK, amikacin; AZM, azithromycin; BDQ, bedaquiline; CAC, co-amoxiclav; CPM, capreomycin; CFZ, clofazimine; CLM, clarithromycin; CYS, cycloserine; EMB, ethambutol; ETO, ethionamide; GFX, gatifloxacin; KNM, kanamycin; LFX, levofloxacin; LZD, linezolid; MFX, moxifloxacin; OFX, ofloxacin; PNH, pasiniasid; PAS, p-aminosalicylic acid; PTO, prothionamide; PZA, pyrazinamide; INH, isoniazid; RPT, rifapentine; RFB, rifabutin; STR, streptomycin; TRD, terizidone; HIV, human immunodeficiency virus; mITT, modified intention-to-treat; N/R, not reported; MDR-TB, multidrug-resistant tuberculosis; XDR-TB, extensively drug-resistant tuberculosis; CI, confidence interval; *This is true for the majority of studies, although some patients in the studies by Xu, 2012 and Misra, 2020 received BDQ, and/or LZD.

### Search strategy

We performed a PubMed database search using the terms “clofazimine,” “B663,” “riminophenazine,” “tuberculosis” and “drug-resistant tuberculosis” with no restrictions, but only considered English language articles for inclusion in this review. Studies with predominantly paediatric populations (<15 years old) were excluded. Reference lists from included publications were reviewed manually to identify any additional relevant publications and data sources.

### History

Clofazimine (formerly B663) was initially described in the mid-1950s as the lead compound in a novel class of antibiotics, the riminophenazines, that showed antituberculosis activity comparable to that of isoniazid in animal studies (Barry et al., 1957). Its discovery was part of a dedicated effort to develop new antituberculosis drugs in the wake of the discovery of streptomycin, para-aminosalicylic acid (PAS) and isoniazid. Clofazimine was derived from a compound called anilinoaposafranine which in turn was synthesized from diploicin, originally extracted from a lichen called *Buellia canescens* (Barry, 1946a; Barry, 1946b; Barry et al., 1956a; Yawalkar and Vischer, 1979). In early studies in mice and hamsters, clofazimine demonstrated impressive activity, including against isoniazid-resistant strains, without evidence of major toxicity (Barry et al., 1957). More limited activity was observed in subsequent guinea pig and primate models (Barry and Conalty, 1965) and further development of clofazimine for the treatment of tuberculosis was halted. These cross-species discrepancies were later speculated to be due to differences in drug absorption, protein binding or pathological manifestations between species (Barry et al., 1960). By the early 1960s, the efficacy of clofazimine against leprosy was demonstrated in human trials (Browne and Hogerzeil, 1962) and clofazimine became a cornerstone of leprosy treatment and is still recommended by the WHO in standard anti-leprosy multidrug therapy today (World Health Organisation, 2018a). Drug repurposing efforts during the 1990s aimed at addressing the rise in DR-TB cases revived interest in the antituberculosis activity of clofazimine (Mehta et al., 1993; Jagannath et al., 1995; Reddy et al., 1996; Adams et al., 1999). In 2010, an observational study conducted in Bangladesh reported 87% treatment success in MDR/RR-TB patients treated with a 9–11 months regimen containing gatifloxacin, an injectable aminoglycoside and clofazimine, with other drugs (Van Deun et al., 2010). This was a substantial improvement over the 50%–60% success rate seen with conventional longer (≥18 months) injection-containing regimens in programmatic settings at the time (Van Deun et al., 2010). Further clinical studies supported the efficacy of the so-called “Bangladesh regimen” in diverse settings, (Nunn et al., 2019; Schwœbel et al., 2019), while preclinical studies also demonstrated a treatment-shortening effect when clofazimine was added to both first- and second-line combination regimens (Grosset et al., 2013; Tyagi et al., 2015; Lee et al., 2017). This led to the widespread off-label use of clofazimine as part of both shorter (≤12 months) and longer (≥18 months) DR-TB regimens. In 2018, when the WHO revised its grouping of drugs for use in individualised DR-TB regimens, clofazimine was re-classified from a Group 5 agent (drugs with unclear significance) to a Group B agent (drugs with second highest priority for use), solidifying its role as a key drug in DR-TB therapy (World Health Organisation, 2018b). In addition to leprosy and tuberculosis treatment, clofazimine is also used in the treatment of some non-tuberculous mycobacteria (Kim et al., 2021) and as an anti-inflammatory agent in certain autoimmune conditions (Arbiser and Moschella, 1995; Gurfinkel et al., 2009). Clofazimine is also being explored for use against Gram-positive bacteria, (Huygens et al., 2005), as an anti-parasitic, (Tuvshintulga et al., 2016; Zhang et al., 2022a), anti-neoplastic (Ahmed et al., 2019; Xu et al., 2020) and anti-viral agent (Zhang et al., 2022b).

### Physicochemical and pharmacokinetic properties

Clofazimine is a cationic, amphiphilic molecule (having both hydrophilic and hydrophobic domains) with extremely high lipophilicity and low aqueous solubility at physiological conditions (Reddy et al., 1999). Its colour varies in a solution depending on the pH, from orange-yellow in alkaline environments to deep red at neutral to mildly acidic pH to violet and eventually colourless in strongly acidic environments (O'Connor et al., 1995). These physicochemical properties contribute to the unusual pharmacokinetics (PK), putative mechanisms of action and adverse effects of clofazimine.

Due to its extremely low aqueous solubility, orally administered clofazimine in coarse crystalline form has low bioavailability with considerable inter-individual variation in absorption kinetics (Barry et al., 1960; Vischer, 1969; Banerjee et al., 1974; Levy, 1974). For this reason, the commercially available preparation (Lamprene^®^, Novartis Pharmaceuticals Corporation) is provided as a micronized (ultra-fine crystal) suspension in an oil-wax base, which improves absorption to around 70% of the administered dose (Vischer, 1969; Yawalkar and Vischer, 1979). Consistent with its high lipophilicity, intake with fatty food improves absorption (Vischer, 1969; Schaad-Lanyi et al., 1987; Nix et al., 2004). The mechanism by which clofazimine crosses from the gastrointestinal tract into circulation is not established, but a fraction is carried in micelles, reaching the systemic circulation *via* the lymph, although this is not thought to be the primary mode of absorption (Barry et al., 1960; O'Connor et al., 1995).

Once absorbed into the systemic circulation, distribution to peripheral compartments occurs rapidly, followed by slow re-equilibration to the central compartment (Schaad-Lanyi et al., 1987), leading to a slow rise in mean plasma concentration and a low steady-state plateau (Schaad-Lanyi et al., 1987; Abdelwahab et al., 2020). A recently published population PK model derived from DR-TB patients demonstrated an extremely large volume of distribution (10,500 L) and long elimination half-life of approximately 30 days, in contrast to previously reported values of ∼10 days (Schaad-Lanyi et al., 1987) and ∼70 days (Levy, 1974) based on observed data from older studies in healthy volunteers and leprosy patients. At 100 mg daily, the standard dose for tuberculosis, simulations from the population PK model showed that steady-state plasma concentrations likely exceed clofazimine’s minimum inhibitory concentration (MIC) for wild-type *Mycobacterium tuberculosis* of 0.25 μg/mL but remain below the critical concentration of 1 μg/mL for resistant strains (World Health Organistation, 2018). As clofazimine is highly protein bound (Irwin et al., 2014; Swanson et al., 2015), the free (unbound) drug fraction is expected to be well below the MIC. One murine PK study suggested that clofazimine’s bactericidal activity is determined by the time plasma concentrations are above MIC (T > MIC) (Swanson et al., 2016), though this *in vivo* exposure-activity relationship has not been confirmed in other studies. In mice receiving clofazimine monotherapy, serum and tissue concentrations were dose- and time-dependent, but bactericidal activity was dose-independent at doses ranging from 6.25 mg/kg to 25 mg/kg (Swanson et al., 2015). In contrast, when clofazimine was added to the standard first-line DS-TB regimen in a mouse model, there was a linear dose-response in terms of bactericidal activity (decline in lung bacterial burden), although there was no difference in the time required to achieve relapse-free cure with the addition of 12.5 mg/kg vs. 25 mg/kg of clofazimine, suggesting the same efficacy can be achieved with the lower of the two doses (Ammerman et al., 2018). Serum concentrations with this dose range in mice are approximately equivalent to that in humans with a 100 mg daily dose, though some inconsistent results in human PK studies mean that uncertainty about dose equivalence remains (Ammerman et al., 2018).

Clofazimine steady-state conditions are reached after several months a consequence of its extended half-life. The use of loading doses shortens time to steady-state, possibly achieving effective concentrations more rapidly, but may increase toxicity related to higher peak exposures. Simulations using the aforementioned population PK model predicted that a loading dose of 200 mg daily for 2–4 weeks, depending on body fat percentage, can shorten time to steady-state by several weeks without increased risk of cardiotoxicity, based on peak concentrations during the loading period not exceeding those at steady-state, and assuming peak concentration correlates with QT-interval prolongation (Abdelwahab et al., 2020). Using joint PK-pharmacodynamic (PD) modelling, a follow-up study predicted that the risk of significant QT-prolongation with a loading dose of 300 mg daily for 2 weeks was no higher than with the standard dose of 100 mg daily (Abdelwahab et al., 2021). Clinical safety of these clofazimine dosing strategies is currently being evaluated in clinical trials. In two separate studies, body fat percentage (which accounted for the significant sex differences in plasma exposures) was identified as an important determinant of clofazimine PK, suggesting an individualized approach may be required for optimal clofazimine dosing (Abdelwahab et al., 2020; Alghamdi et al., 2020).

In contrast to the low concentrations detected in plasma, massive duration-dependent accumulation of clofazimine occurs in tissues, particularly in adipose tissue and macrophage-rich organs such as the spleen, liver, lungs, gut and lymph nodes (Mansfield, 1974; Baik et al., 2013; Swanson et al., 2015). The mechanisms by which clofazimine crosses cellular membranes and the selective intra-macrophage accumulation are not fully understood, but an active transport mechanism rather than *via* passive diffusion has been hypothesized (O'Connor et al., 1995). PK studies in mice suggest that tissue accumulation of clofazimine occurs in two phases: initially, the highest concentrations are observed in fat, in keeping with passive, concentration-dependent partitioning of a highly lipophilic molecule. Later, drug concentrations in the liver, spleen, lungs and other macrophage-rich organs greatly exceed concentrations in fat (Vischer, 1969; Baik et al., 2013; Keswani et al., 2015). Biopsies of these organs display crystal-like structures of sequestrated clofazimine found exclusively inside macrophages (Baik and Rosania, 2011; Baik et al., 2013). These solid drug aggregates, known as crystal-like drug inclusions (CLDI), contain a hydrochloride salt form of clofazimine and are responsible for the blackish discolouration of macrophage-rich internal organs (Baik and Rosania, 2012; Baik et al., 2013; Murashov et al., 2018a). CLDI formation appears to be an intracellular process related to the lysosomal microenvironment inside macrophages (which have low pH and high chloride concentrations) rather than extracellular precipitation and phagocytosis of drug crystals (Baik and Rosania, 2012; Baik et al., 2013). The tendency of clofazimine to concentrate inside macrophages was recognized early on and was initially viewed as a favourable characteristic, considered to be a form of targeted drug delivery for intracellular pathogens such as *M. tuberculosis and M. leprae* (Conalty et al., 1971). Currently, however, the activity of this large pool of sequestrated drug inside macrophages is less clear. Since this stable, intracellular drug pool in CLDI gets released during *ex vivo* processing, the high concentrations of clofazimine measured in homogenised tissue samples are likely misleading and may have limited value in predicting the exposure-response relationship of clofazimine. Studies of resected lung tissue from DR-TB patients who underwent therapeutic lung resection following clofazimine treatment demonstrated that clofazimine accumulates in the outer cellular layers of granulomas and cavity walls, but penetrates poorly into the acellular, necrotic centre of caseous lesions, further complicating the relationship between tissue concentrations and drug activity (Prideaux et al., 2015; Strydom et al., 2019). Notwithstanding these difficulties with interpreting tissue concentrations, drug accumulation in macrophages and specific tissues likely increases site-of-disease concentrations thereby contributing to the efficacy of clofazimine.

The mechanisms involved in clofazimine in metabolism and excretion are not fully established. The amount of clofazimine excreted unchanged in the urine is negligible (Banerjee et al., 1974; Levy, 1974). Three urinary metabolites of clofazimine, also present in negligible concentrations, has been described (Feng et al., 1981; Feng et al., 1982). In contrast, a relatively large but variable proportion of orally administered clofazimine can be recovered unchanged in the faeces (Banerjee et al., 1974; Levy, 1974). It is unclear if faecal excretion represents incomplete absorption from the gut or biliary excretion, as high levels of clofazimine have been found in the bile and gall bladder in an autopsy study (Mansfield, 1974). Small quantities of clofazimine are also excreted in sweat, sputum, lacrimal fluid, sebum and breastmilk (Vischer, 1969; Venkatesan et al., 1997; Novartis Pharmaceuticals Corporation, 2019). Clofazimine is at least partially metabolised in the liver. An *in vitro* study using human liver microsomes identified eight metabolites of clofazimine as well as the enzymatic pathways involved in their formation, including the important cytochrome P450 isoenzymes CYP3A4/A5 and CYP1A2 (Howlader et al., 2022). In HepaRG cells, clofazimine was a weak inducer of CYP3A4 at low concentrations, but inhibited CYP3A4 at therapeutic concentrations, suggesting a degree of auto-induction and the potential for clinically significant interactions with drugs metabolized by CYP3A4 (Horita and Doi, 2014; Shimokawa et al., 2015). However, one study among DR-TB patients did not find a significant difference in clearance of bedaquiline (a CYP3A4 substrate) or its M2 metabolite when co-administered with or without clofazimine (Maartens et al., 2018). Clofazimine tissue concentrations are not affected by co-administration with rifampicin, a strong inducer of CYP3A4 (Mamidi et al., 1995), while co-administration with isoniazid produces increased plasma and lung concentration but reduced concentrations in several other tissues (Venkatesan et al., 2007; Lu et al., 2009). In light of the limited evidence, current guidelines do not recommend dose adjustment of clofazimine or specific companion drugs during co-administration. The manufacturer’s package insert advises caution when using clofazimine in patients with liver impairment but no need for dose adjustment with mild to moderate renal impairment (Novartis Pharmaceuticals Corporation, 2019), though published literature on use in these scenarios could not be found.

Because of a tendency to accumulate in fatty tissue, clofazimine is likely to equilibrate rapidly into brain tissue and may have therapeutic potential for neurological TB. Clofazimine was undetectable in cerebrospinal fluid (CSF) from patients with tuberculous meningitis (TBM) (Kempker et al., 2022) and brain tissue in autopsy studies from leprosy patients (Mansfield, 1974; Desikan and Balakrishnan, 1976). This is likely a result of extensive protein binding with extremely low concentrations of free drug equilibrating into the central nervous system from plasma; clofazimine concentrations in this compartment may be below the limit of detection of older assays and therefore may not reflect a true absence of drug. Supporting this, time-dependent tissue concentrations and widespread spatial distribution of clofazimine were demonstrated by mass spectrometry imaging throughout the brain in mice at a dose of 100 mg/kg (several-fold the therapeutic dose for tuberculosis) (Baijnath et al., 2015). At the same high dose, monotherapy with clofazimine but not linezolid was able to completely prevent central nervous system dissemination of *M.tb* after aerosol infection of mice (Baijnath et al., 2018). Case reports exist of successful treatment of patients with drug-resistant TBM using clofazimine in combination with other new and repurposed second-line agents (Tucker et al., 2019).

### Mechanism of action

**FIGURE 1:**
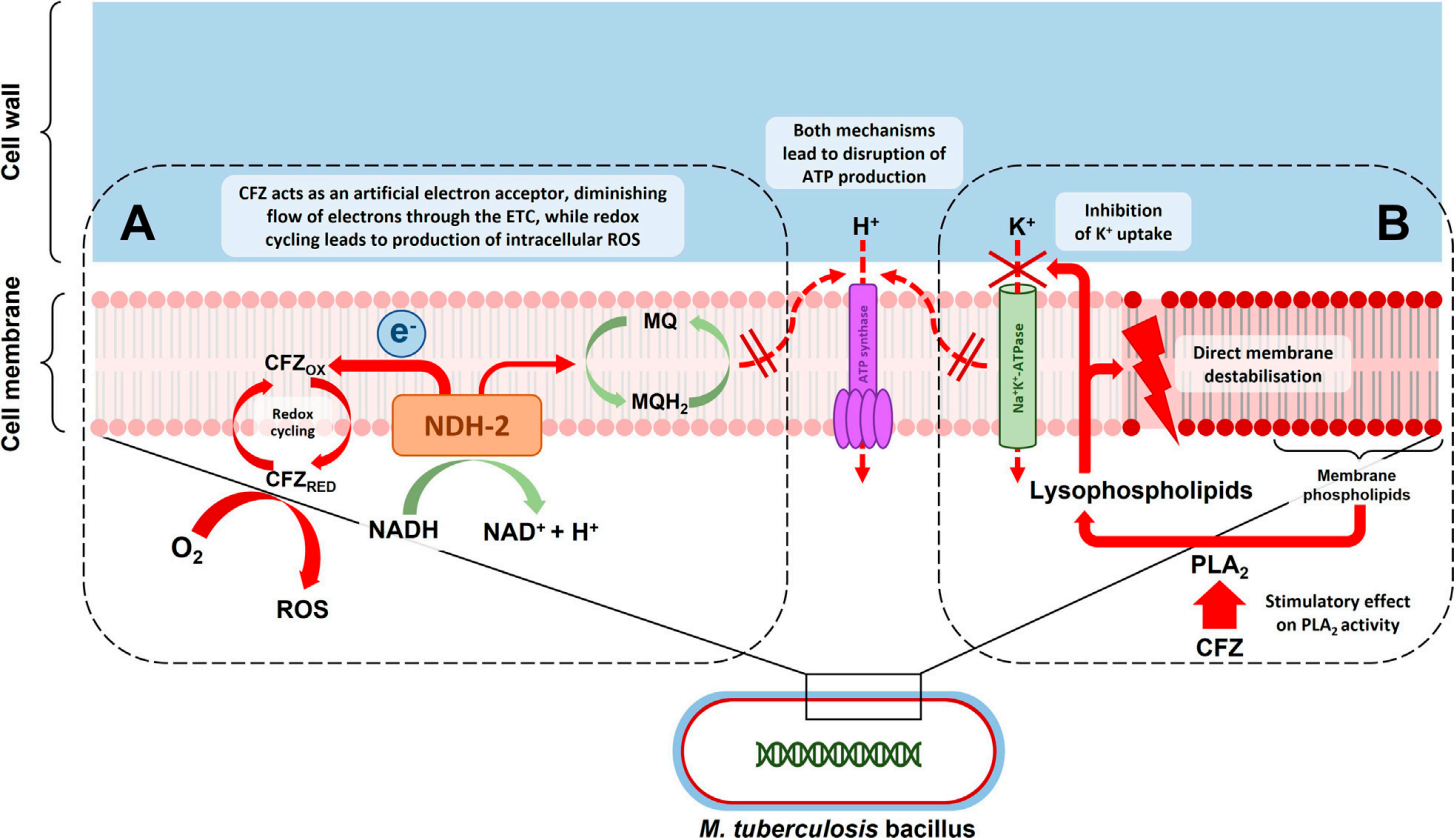
Depiction of the putative mechanisms of action of clofazimine (CFZ) acting at the level of the mycobacterial cell membrane: **(A)** CFZ competes with menaquinone (MQ) as a substrate of type 2 NADH dehydrogenase (NDH-2) in the first step of the mycobacterial electron transport chain (ETC). This draws electrons away from the ETC, possibly reducing ATP production. Reduced CFZ produced in the process is spontaneously re-oxidized in the presence of intracellular oxygen (O_2_), leading to the formation of intracellular reactive oxygen species (ROS); **(B)** Clofazimine leads to an increase in lysophospholipids in a process mediated by phospholipase A_2_ (PLA_2_) activity. Lysophospholipids inhibit potassium (K^+^) uptake and cause direct membrane destabilisation, thereby also disrupting ATP production.

Clofazimine’s exact mechanism of action against *M. tuberculosis* is not completely understood, but its primary actions are thought to occur at the level of cellular membranes, likely interfering with membrane-associated physiological processes including cellular respiration and ion transport (Cholo et al., 2017). This is depicted in Figure 1. Barry et al. who originally described the antituberculosis activity of clofazimine noted the redox properties of the compound and proposed a mechanism of action whereby redox cycling of clofazimine contributed to growth inhibition and cell death either through the production of intracellular oxygen radicals or partial inhibition of cellular respiration or a combination of these effects (Barry et al., 1956b). A biochemical pathway supporting this hypothesis was later described whereby clofazimine competes with menaquinone as substrate of the respiratory chain enzyme NDH-2, acting as an artificial electron acceptor (Yano et al., 2011; Lechartier and Cole, 2015), thereby shunting electrons away from the respiratory chain and ultimately decreasing adenosine triphosphate (ATP) production. It was also shown that the reduced clofazimine produced through this process is spontaneously re-oxidized in the presence of oxygen, leading to the formation of intracellular reactive oxygen species (Yano et al., 2011). Initially thought to be an NDH-2-dependent process (Yano et al., 2011), it has since been demonstrated that the bactericidal activity of clofazimine in *M. tuberculosis* does not require NDH-2 (Beites et al., 2019). Others have questioned whether this redox mechanism is clofazimine’s primary mode of action and have instead produced evidence, based on studies in Gram-positive bacterial organisms, that the bactericidal activity of clofazimine is related to stimulation of phospholipase A_2_ activity and production of toxic lysophospholipids which disrupt transmembrane potassium transport (Van Rensburg et al., 1992; Steel et al., 1999). Other proposed mechanisms that may contribute to clofazimine’s bactericidal action include i) direct, non-specific membrane disruption (Oliva et al., 2004), ii) direct interference with bacterial potassium uptake (De Bruyn et al., 1996; Steel et al., 1999), iii) selective binding to mycobacterial DNA with blocking of template function (Morrison and Marley, 1976a; Morrison and Marley, 1976b) and iv) reversal of the inhibitory effects of certain mycobacterial proteins on phagocyte activity (Wadee et al., 1988). In summary, clofazimine appears to have multiple mechanisms of antimicrobial activity, possibly with differential importance of specific mechanisms under distinct physiological conditions (Lu et al., 2011; Cholo et al., 2017), which may explain the lack of a single dominant, target-specific genetic marker associated with clofazimine resistance (CRyPTIC Consortium, 2022).

### Clofazimine resistance

The selection of clofazimine-resistant *M. tuberculosis* isolates has been demonstrated *in vitro* (Hartkoorn et al., 2014) and reported in clinical isolates (Xu et al., 2017; Nimmo et al., 2020a). The MIC distribution of clofazimine in mycobacterial growth indicator tube (MGIT) culture systems ranges between 0.125 µg/mL-0.5 μg/mL for pan-susceptible and 0.25 µg/mL-1 µg/mL for DR-TB strains (Ismail et al., 2019), with a critical concentration of 1 μg/mL recommended by the WHO (World Health Organistation, 2018). Reliable estimates of the prevalence of clofazimine resistance are not available. In a large multi-national data set of over 12,000 isolates compiled by the CRyPTIC Consortium to study genotype-phenotype associations (55% had resistance to at least one antituberculosis drug), the prevalence of phenotypic clofazimine resistance was 4.4% overall and 41% (59/142) in extensively drug-resistant isolates (XDR; resistance to rifampicin, isoniazid, fluoroquinolones and another WHO Group A drug i.e. bedaquiline or linezolid) (Consortium, 2022). A recent Korean study of 122 MDR- and XDR-TB isolates reported a 4% prevalence of phenotypic clofazimine resistance (Park et al., 2022). Two studies from South Africa examined the presence of resistance-associated variants (RAVs) in the Rv0678 gene (Rv0678 RAVs are associated with phenotypic bedaquiline resistance and cross-resistance to clofazimine) in clinical DR-TB isolates with varying exposure to clofazimine or bedaquiline (Nimmo et al., 2020a; Nimmo et al., 2020b). Rv0678 RAVs were detected in 1.8% (7/391) and 5.4% (5/92) of pre-treatment isolates, while treatment-emergent Rv0678 RAVs occurred in 2% (8/392) and 5.7% (5/87), respectively. Although these reports give some indication of the frequency of clofazimine resistance observed in relatively large, pooled sets of clinical DR-TB isolates, they do not represent accurate prevalence estimates for the general DR-TB population or specific sub-groups due to the heterogenous sampling methodology used. For example, the sampling methodology for the CRyPTIC data set was biased towards collecting resistant isolates with temporally and geographically matched susceptibles wherever possible and differed markedly between contributing sites/countries, while the country-specific reports used pooled samples contributed by multiple primary studies with diverse eligibility criteria conducted at specialized DR-TB treatment centres.

Much uncertainty still exists regarding the genetic basis of clofazimine resistance, though higher clofazimine MICs have been associated with mutations in several genes including Rv0678, Rv 1979c and Rv2535c (*pepQ)* (Zhang et al., 2015a; Almeida et al., 2016). The report by the CRyPTIC Consortium evaluated genotype-phenotype associations using whole genome sequencing and quantitative MIC data for these and other resistance-associated genes (Rv3249c, Rv 1816, mmpL5, mmpS5, mmpL3), but concluded that no single gene or small group of genes fully explains a substantial proportion of clofazimine resistance, indicating that the significance of all these genes needs further evaluation to clarify their potential role as diagnostic markers (Consortium, 2022). The resistance mechanisms involved appear not to be target-based with some genes associated with MIC elevations of more than one drug (Consortium, 2022). In this regard, bedaquiline cross-resistance is of particular concern and appears to be largely due to mutations in Rv0678 (Nimmo et al., 2020a), although Rv 1979c and *pepQ* have also been associated with low-level bedaquiline cross-resistance (Zhang et al., 2015a; Almeida et al., 2016). Rv0678 is a transcriptional repressor of MmpL5 and MmpS5 efflux pumps (Hartkoorn et al., 2014). Loss of function mutations in this gene are associated with a 2- to 4-fold rise in clofazimine MIC (Andries et al., 2014; Zhang et al., 2015a) and confer cross-resistance to bedaquiline and azole antifungal drugs (Hartkoorn et al., 2014), presumably due to over-expression of these multi-substrate efflux pumps leading to decreased intracellular concentrations of these drugs. Although cross-resistance can be selected for by exposure to any of these drugs (Hartkoorn et al., 2014), bedaquiline resistance seems to more strongly predict clofazimine cross-resistance than the converse. In the multinational CRyPTIC data set, 52.4% of bedaquiline-resistant isolates were also resistant to clofazimine, compared to only 10.6% of clofazimine-resistant isolates having cross-resistance to bedaquiline (Consortium, 2022). In another study, 100% (9/9) bedaquiline-resistant isolates were found to also be resistant to clofazimine with almost all of these (8/9) harbouring Rv0678 RAVs, but only 30% (9/30) of clofazimine resistant isolates had bedaquiline cross-resistance (Ismail et al., 2018). While these results may mean that the bulk of clofazimine resistance currently is not due to Rv0678 mutations, the observation that Rv0678-associated bedaquiline resistance strongly predicts clofazimine resistance means this picture may change over time with increasing use of bedaquiline. Adding to this concern is the long eminination half-lives of both clofazimine and bedaquiline; treatment lapses with regimens containing either of these drugs may expose remaining viable bacilli to low concentrations without companion drugs for protracted periods, thereby creating a high-risk scenario for selection of resistant and cross-resistant variants. For this reason, given their key role in DR-TB treatment, surveillance capacity for both bedaquiline and clofazimine resistance should be an important pillar of the programmatic use of these drugs. No studies were found that assessed the impact of baseline or treatment-emergent clofazimine resistance on clinical or bacteriological outcomes in the context of bedaquiline-containing multidrug regimens and this warrants further study.

### Activity against *M. tuberculosis*

In preclinical studies (*in vitro*, intracellular and different mouse models), clofazimine monotherapy demonstrated bactericidal activity against *M. tuberculosis* similar to that of rifampicin and isoniazid, and importantly, this activity is preserved against strains resistant to these two key first-line antituberculosis drugs (Jagannath et al., 1995; Reddy et al., 1996; Xu et al., 2012a). In mice, monotherapy with doses ranging from 6.25 mg/kg-25 mg/kg does not display early bactericidal activity (EBA; first 7–14 days of treatment) but dose-independent bactericidal activity is evident with longer exposure (Swanson et al., 2015; Ammerman et al., 2017). This lack of EBA has also been demonstrated in a phase 1 trial in patients with tuberculosis (Diacon et al., 2015). Due to the slow elimination of clofazimine, antimicrobial activity is maintained for weeks after treatment cessation, depending on the duration of administration, possibly contributing to the treatment-shortening effect associated with its use (Swanson et al., 2015; Swanson et al., 2016). Clofazimine has potent bactericidal activity against slowly-replicating persister forms of *M. tuberculosis* using diffferent *in vivo* models and a streptomycin-starved *M. tuberculosis* 18b strain infection model in mice (Cho et al., 2007; Zhang et al., 2012a; Khan et al., 2019). The ability to target these drug-tolerant subpopulations is another factor thought to play a role in its treatment-shortening potential. In contrast, clofazimine has been shown to have limited activity in the Kramnik mouse model that exhibits human-like large, caseous granuloma formation in the lungs (Irwin et al., 2014). Clofazimine was also found to be ineffective *in vitro* against biofilm-encased, non-replicating *M. tuberculosis* (Mothiba et al., 2015). It is unclear if this lack of activity is due to a lack of drug penetration to the bacilli in these experimental conditions or the dormant physiological state of the organism under such hypoxic microenvironments. These findings highlight the need for clofazimine to be used as part of combination regimens able to target *M. tuberculosis* in the diverse infection sites and physiological states present in the human host. Clofazimine exhibits additive or synergistic activity with several first- and second-line antituberculosis drugs and has been shown to shorten the time required to achieve relapse-free cure in mice by several weeks when added to both first- and second-line multidrug combinations (Williams et al., 2012; Grosset et al., 2013; Zhang et al., 2015b; Lopez-Gavin et al., 2015; Tyagi et al., 2015; Saini et al., 2019). In particular, the combination of clofazimine, bedaquiline and pyrazinamide has been consistently shown to be a synergistic and highly potent combination in the mouse model (Tasneen et al., 2011; Williams et al., 2012; Silva et al., 2016). A dose-optimized version of this combination, either alone or combined with a fourth companion drug shortens the time required to achieve relapse-free cure in mice by 75%–80%, from 16 weeks to just 3–4 weeks in Kramnik and BALB/c mouse models (Lee et al., 2017; Lee et al., 2018; Clemens et al., 2019). This regimen is currently being advanced into phase II clinical trials to assess its treatment-shortening effect for patients with DS-TB.

In humans, randomized trials evaluating the additive effect of clofazimine for DR-TB treatment are limited to four small-scale trials (49–140 total participants), all conducted in China, that compared clofazimine added to the local standard of care against the standard of care alone (Tang et al., 2015; Wang et al., 2018; Duan et al., 2019; Du et al., 2020). The standard of care regimens in all of these trials consisted of long (≥18 months), injectable-containing regimens that excluded bedaquiline and other new or repurposed drugs. None of these trials included HIV-positive patients, only one trial included patients with XDR-TB and there was no follow-up to assess for relapse or death after treatment completion. In the three trials involving only MDR-TB patients (Tang et al., 2015; Duan et al., 2019; Du et al., 2020), treatment success (the sum of the outcomes “treatment completed” or “cured”) in the clofazimine arm ranged between 65.1% and 73.6% and was significantly higher than the control arm in two of the trials. Time to sputum culture conversion was also significantly shorter in the clofazimine arms in these two trials (Tang et al., 2015; Duan et al., 2019), while the other trial showed no difference (Du et al., 2020). The trial in XDR patients did not find a significant difference between arms for either treatment success (36.3% in the clofazimine group vs. 44.4% in the control group) or time to sputum culture conversion (Wang et al., 2018). In a meta-analysis, pooled results of these four trials favoured the clofazimine group with a higher probability of treatment success overall (relative risk (RR): 1.2, 95% confidence interval (CI): 1.0–1.4, *p* = 0.020) and a lower risk of treatment failure (RR: 0.5, 95% CI: 0.5–0.6, *p* < 0.001), but no difference in mortality (Wang et al., 2022). Key characteristics and results of randomized trials and observational studies evaluating the safety and efficacy of clofazimine-containing regimens (without the Group A drugs bedaqualine and linezolid) are summarised in Table 2. Though most observational studies lacked clofazimine-free controls for direct comparison, the treatment success rate in studies of shorter regimens based on the one used by Van Deun et al. in Bangladesh (Van Deun et al., 2010) ranged between 78.8% and 89.3% amongst MDR-TB patients, despite the absence of bedaqualine or linezolid (Aung et al., 2014; Piubello et al., 2014; Kuaban et al., 2015; Trebucq et al., 2018; Nunn et al., 2019). Furthermore, in the original “Bangladesh study” the group receiving clofazimine for the full duration of treatment achieved 87% treatment success compared to 66% in those receiving clofazimine during the intensive phase only (Van Deun et al., 2010). In an individual patient data meta-analysis of observational studies combining records of over 12,000 DR-TB patients from 25 countries, 824 of whom received clofazimine, the use of clofazimine (compared to non-use) was associated with a higher probability of treatment success in pooled analysis (adjusted risk difference (aRD): 0.06, 95% CI: 0.01–0.10) and in the XDR-TB subgroup, its use was associated with reduced mortality (aRD: −0.18, 95% CI: −0.27 to −0.10), but not increased treatment success (Ahmad et al., 2018).

**TABLE 2:** Ongoing and recently completed clinical trials evaluating clofazimine-containing shorter regimens for drug-susceptible and -resistant tuberculosis.

Trial	Phase	Target population; sample size	Country	Study design and regimens	Primary efficacy outcome
Drug-susceptible tuberculosis					
NCT03474198 (TRUNCATE-TB)	Phase 2/3	DS-TB; 900 (180 per arm)	Multi-country (Asia)	Randomized, open-label, multi-arm, multi-stage trial comparing four experimental 2–3 month regimens (one CFZ-containing) *versus* 6-month standard of care for drug-susceptible tuberculosis	Unfavourable clinical outcome 96 weeks after randomisation
NCT04311502 (CLO-FAST)	Phase 2	DS-TB; 185	Multi-country	Randomized, open-label trial comparing a 3-month RPT/CFZ-containing regimen with CFZ loading dose *versus* 6-month standard of care for drug-susceptible tuberculosis	Time to stable culture conversion in liquid media through 12 weeks
NCT05556746 (PRESCIENT)	Phase 2	DS-TB; 156	South Africa, Haiti	Randomized, open-label trial comparing an 8-week regimen of BDQ, CFZ, PZA, and DLM with standard treatment for drug-susceptible pulmonary tuberculosis	Time to stable culture conversion in liquid media through 8 weeks
Drug-resistant tuberculosis					
NCT04545788	Phase 2	MDR/RR-TB; 200	China	Randomized, open-label, multi-arm trial comparing two fully oral 9–11 month experimental regimens for rifampicin-resistant tuberculosis *versus* standard of care (9–11 months injectable-containing regimen. (All regimens contain CFZ.)	Sputum culture conversion and clinical outcomes (not otherwise specified)
NCT02589782 (TB-PRACTECAL)	Phase 2/3	MDR/RR-TB, pre-XDR-TB; 552	South Africa, Belarus, Uzbekistan	Randomised, open label, multi-arm phase II-III trial evaluating short regimens containing BDQ and PA in combination with existing and re-purposed anti-TB drugs (LZD, MFZ and CFZ) for MDR-TB, irrespective of fluoroquinolone resistance	Percentage of patients with an unfavourable outcome (failure, death, recurrence, loss to follow-up) at week 72 after randomisation
NCT03828201 (DRAMATIC)	Phase 2	MDR/RR-TB; 220	Vietnam, Philippines	Multicentre, randomized, partially blinded, four-arm, phase 2 study examining the efficacy and safety of an all-oral regimen of BDQ, DLM, LFX, LZD, and CFZ for 16, 24, 32 or 40 weeks	Favourable clinical outcome (“treatment success”) 76 weeks after randomisation
NCT04062201 (BEAT-TB)	Phase 3	MDR/RR-TB, pre-XDR-TB, XDR-TB; 402	South Africa	Open-label, multi-centre, randomized controlled trial comparing a 6-month regimen of BDQ, DLM, LZD, LFX and CFZ *versus* the local standard of care in South Africa (9 months)	Proportion of participants with a successful outcome at the end of treatment and at week 76
NCT03867136 (TB-TRUST)	Phase 3	MDR-TB; 354	China	Multicentre, open-label, randomized controlled trial comparing a short (24–44 weeks) all-oral regimen consisting of LFX, LZD, CYS and PZA and/or CFZ, guided by PZA susceptibility testing, *versus* the WHO standardized shorter regimen for MDR-TB (36–44 weeks)	Proportion of participants with a successful outcome 84 weeks after randomisation
TB-TRUSTplus (NCT04717908)	Phase 3	pre-XDR; 200	China	Multicentre, open-label trial evaluating a short (24–44 weeks) all-oral regimen consisting of BDQ, LZD, CYS, PZA and/or CFZ, guided by PZA susceptibility testing	Proportion of participants with a successful outcome 84 weeks after randomisation
NCT05278988	Phase 2	MDR-TB; 60	China	Randomized, open-label trial comparing a shorter (6–9 months) all-oral regimen containing BDQ, DLM, PZA and CFZ *versus* the WHO standard of care for MDR-TB (9–11 months)	Clinical outcomes at study end (18 months follow-up), not otherwise specified
NCT02754765 (endTB)	Phase 3	MDR/RR-TB; 754	Multi-country	Randomized, controlled, open-label, non-inferiority, multi-country trial evaluating the efficacy and safety of five new, all-oral, shortened regimens (three CFZ-containing) for multidrug-resistant tuberculosis (MDR-TB)	Proportion of participants with favourable outcome at week 73 after randomisation
NCT03896685 (endTB-Q)	Phase 3	pre-XDR; 324	Multi-country	Randomized, controlled, open-label, non-inferiority, multi-country trial evaluating the efficacy and safety of two new, all-oral, shortened regimens for multidrug-resistant tuberculosis (MDR-TB) with fluoroquinolone resistance	Proportion of participants with favourable outcome at Week 73 randomisation
NCT05306223 (PROSPECT)	Phase 3	MDR/RR-TB; 212	China	Pragmatic, randomized, controlled trial comparing two oral short regimens (both containing CFZ) for MDR-TB.	Proportion of participants with favourable outcome at the end of treatment (week 40)

Abbreviations: BDQ, bedaquiline; CFZ, clofazimine; CYS, cycloserine; DLM, delamanid; EMB, ethambutol; LFX, levofloxacin; LZD, linezolid; MFX, moxifloxacin; PA, pretomanid; PZA, pyrazinamide; RPT, rifapentine; RFB, rifabutin; MDR-TB, multidrug-resistant tuberculosis; XDR-TB, extensively drug resistant tuberculosis; WHO, world health organisation.

### Safety and tolerability

An important factor in the use of clofazimine for DR-TB treatment is its favourable tolerability profile compared with other second-line antituberculosis drugs. Although serious gastrointestinal complications have been reported in leprosy patients after prolonged, high-dose clofazimine treatment (McDougall et al., 1980; Chong and Ti, 1993; Singh et al., 2013), adverse events requiring interruption or cessation of the drug are infrequently reported in the tuberculosis treatment literature. (For leprosy, the WHO currently recommends a dose of 50 mg daily plus 300 mg monthly for 6–12 months (World Health Organisation, 2018c), though much higher doses and longer durations have been used for leprosy in the past and for anti-inflammatory indications e.g. 300 mg–400 mg daily to treat erythema nodosum leprosum and pyoderma gangrenosum.) (Yawalkar and Vischer, 1979; Arbiser and Moschella, 1995) The most concerning and common adverse events attributed to clofazimine are discolouration of the skin, gastrointestinal disturbances and QT interval prolongation.

Reddish-brown or blackish skin discolouration is commonly reported in patients receiving long-term clofazimine therapy for tuberculosis (Xu et al., 2012b; Tang et al., 2015; Dalcolmo et al., 2017). More pronounced skin discolouration in sun-exposed areas is reported, and phototoxicity is listed as a rare adverse effect in the manufacturer’s prescribing information (Hastings et al., 1976; Yawalkar and Vischer, 1979; Novartis Pharmaceuticals Corporation, 2019). However, some larger studies reporting on skin discolouration do not seem to confirm this observation (Browne, 1965; Schulz, 1971; Moore, 1983) and a definite link between sun exposure and the severity of clofazimine-induced skin discolouration is unconfirmed. Other skin symptoms frequently reported with clofazimine include ichthyosis and pruritis (Moore, 1983; Xu et al., 2012b). Discolouration of conjunctivae, sclerae, mucosa, urine, faeces and sweat has also been reported in leprosy patients (Browne, 1965; Moore, 1983; Kumar et al., 1987). Incidence of skin discolouration in tuberculosis patients varies widely, ranging between 10% and 94% (Xu et al., 2012b; Tang et al., 2015; Dalcolmo et al., 2017; Wang et al., 2018; Duan et al., 2019; Misra et al., 2019) which may in part be explained by variation in constitutional skin pigmentation between different study populations, differences in treatment dose and duration, and the lack of a standardized definition and objective measurement methodology of skin discoloration. Two distinct discolouration phenomena have been recognized: the first type is an early onset, more subtle and generalized reddish discolouration while the second type occurs later in therapy as dark brown or blackish hyperpigmentation that is more localized and differentially affects the inflammatory lesions found in leprosy patients (Browne, 1965; Levy and Randall, 1970; Job et al., 1990; Bishnoi et al., 2019). Generalized, reddish discolouration also occurs in mice exposed to clofazimine resulting from partitioning of free clofazimine into subcutaneous fat and skin tissue, rather than the hydrochloride salt form found within macrophages containing CLDI (Murashov et al., 2018a). However, skin biopsies from hyperpigmented inflammatory lesions in leprosy patients have confirmed lesional infiltration by foamy macrophages containing both clofazimine CLDI and ceroid lipofuscin pigment aggregates, both of which can contribute to the characteristic darkening of these lesions (Job et al., 1990; Bishnoi et al., 2019). The onset of visible skin discolouration can become noticeable within days to weeks and typically takes several months to resolve after treatment cessation, but detailed time course data are limited (Browne, 1965; Moore, 1983). Subjectively judged severity/intensity of skin and organ discoloration has been reported to correlate with treatment dose and duration in animal studies (Swanson et al., 2015), but this has not been objectively quantified or related to plasma drug concentrations. One retrospective review of clofazimine toxicity among DR-TB patients in South Africa did not find a statistically significant difference in the occurrence of skin discolouration between different dose-weight categories (Misra et al., 2019). Skin discolouration is not a frequent reason for interruption or cessation of clofazimine therapy by treating clinicians as the condition is largely considered a cosmetic rather than toxicity problem (Moore, 1983; Dalcolmo et al., 2017). However, in one study, patients described skin changes as “stigmatizing” (Ramu and Iyer, 1976) and case reports of depression have been linked to skin discolouration (Tang et al., 2015), illustrating the adverse impact it may have on patients’ quality of life. In a recent trial conducted in China evaluating alternative short all-oral regimens that factored in drug affordability and patients’ willingness to tolerate skin discolouration, more than 10% of patients opted out of clofazimine treatment when given the option (Fu et al., 2021). These reports suggest that clofazimine-induced skin discoloration may significantly impact on quality of life and psychological wellbeing in some patients or populations and may ultimately affect willingness to adhere to treatment, if ignored.

As with skin, multiple reports exit of brownish discoloration with crystalline deposits affecting the conjunctiva, cornea, and lacrimal fluid of patients (Ohman and Wahlberg, 1975; Wålinder et al., 1976; Nêgrel et al., 1984; Barot et al., 2011). Clofazimine’s package insert includes a warning of associated dimness of vision, burning, and itching of eyes (Novartis Pharmaceuticals Corporation, 2019), though in many cases eye discolouration appears to be asymptomatic (Wålinder et al., 1976; Nêgrel et al., 1984). Clofazimine has also been associated with retinal degeneration, though cytomegalovirus could not be excluded as a possible contributing cause in these patients with advanced HIV disease (Craythorn et al., 1986; Cunningham et al., 1990). Ocular complications of clofazimine tend to be associated with higher doses given for anti-inflammatory indications or during very prolonged treatment in patients with leprosy (Wålinder et al., 1976; Nêgrel et al., 1984; Barot et al., 2011), and reports of eye complications is rare in the tuberculosis treatment literature. Like skin discolouration, most eye changes improve over the course of months once clofazimine is stopped (Wålinder et al., 1976; Barot et al., 2011).

Cardiac safety concerns associated with clofazimine are based on several lines of evidence. A case report of *torsade de pointes* in a leprosy patient which occurred after prolonged, high-dose clofazimine treatment identified the drug as the most likely cause of the arrhythmia after the exclusion of other causes (Choudhri et al., 1995). Clofazimine strongly inhibits hERG cardiac potassium channels (Wallis, 2016), which results in QT-prolongation, with potential for ventricular arrhythmias and sudden cardiac death (Chen et al., 1999; Lehmann et al., 2018). Corrected QT-interval (QTc) prolongation ≥ 500 ms has been shown to correlate with an increased risk of *torsade de pointes* (Li and Ramos, 2017). In a 14-day EBA study in DS-TB patients, clofazimine monotherapy produced a duration-dependent increase from baseline in corrected QT-interval (ΔQTc) that was higher than in study arms not including any QT-prolonging drugs (Diacon et al., 2015). Though the QTc increase in this study was modest (range: 16–20 ms), clofazimine exposure was not yet at steady-state due to the short study duration. Using data from the same study, modeling and simulation of the concentration-QTc relationship predicted a mean QTc increase at steady-state of 28.5 ms with a clofazimine dose of 100 mg daily, which is higher than the values reported for the other commonly used QT-prolonging DR-TB drugs moxifloxacin, bedaquiline and delamanid (Abdelwahab et al., 2021). An important concern is a potential for additive cardiotoxicity when clofazimine is co-administered with other QT-prolonging drugs, as is the case in current WHO-recommended DR-TB regimens (World Health Organisation, 2022b). In the EBA trial, an increase from pretreatment baseline (ΔQTc) value ≥ 60 ms was noted in 7% (1/15) of patients receiving clofazimine alone and 27% (4/15) of patients receiving clofazimine, bedaquiline and pretomanid combined (Diacon et al., 2015). A phase 2 bedaquiline trial reported a mean maximum change from baseline in QTc (ΔQTc_max_) of 12.3 ms in those receiving bedaquiline alone compared to 31.9 ms in those taking bedaquiline plus clofazimine (Pym et al., 2016). In the STREAM-1 trial, the median ΔQTc_max_ was 50 ms (IQR: 36-66) in the intervention arm containing clofazimine plus high-dose moxifloxacin compared to 30 ms (IQR: 22-41) in the control arm containing a fluoroquinolone without clofazimine (Hughes et al., 2022). The rate at which a QTc ≥ 500 ms occurred was also higher in the intervention than the control arm (hazard ratio: 2.3, 95% CI: 1.0–5.3) and the proportion of patients who developed a QTc ≥ 500 ms was numerically higher in those receiving clofazimine, although the difference was not statistically significant (11% vs. 6.4%, *p* = 0.14). Four cases of sudden death were reported in the trial, although only one in each arm was attributed to tuberculosis treatment and not explicitly linked to QT-prolongation. In the same trial, having a QTc of ≥ 400 ms at baseline was predictive for developing a QTc ≥ 500 ms, while the per kilogram dose of clofazimine and moxifloxacin was not (Hughes et al., 2022). Optimised clofazimine dosing strategies, specifically the use of loading doses, should take these cardiac safety concerns into consideration. As discussed earlier, a PK-PD simulation showed that a loading dose of 300 mg daily for 2 weeks may not increase the risk of severe QT prolongation while reducing the time to steady state (Abdelwahab et al., 2021). In this study the predicted proportion with a ΔQTc increase >30 ms from baseline by the end of the loading period was 31%, compared with 33% at steady-state with either the standard 100 mg daily or loading dose regimen and the proportion with an absolute QTc >450 ms was 3.4% at steady state with eitherdosing strategy. In view of these findings, regular electrocardiographic monitoring is recommended with clofazimine-containing regimens, especially when combined with other QT-prolonging drugs.

Gastrointestinal upset (including anorexia, nausea and vomiting, diarrhoea and abdominal pain) is frequently reported as a clofazimine-related adverse event in the leprosy literature and is particularly linked to the use of higher doses for anti-inflammatoryindications (Jopling, 1976). In some cases, abdominal pain was severe enough to warrant laparotomy (Jopling, 1976; Chong and Ti, 1993; Sukpanichnant et al., 2000). The causal role of clofazimine in these symptoms was based on a temporal association between symptom onset and start of treatment, the resolution of symptoms after decreasing or withdrawing the drug and exclusion of other obvious causes for the symptoms (Jopling, 1976). With DR-TB treatment, gastrointestinal intolerance appears to be less of an issue than with leprosy treatment, possibly due to the lower doses used for tuberculosis. Gastrointestinal symptoms have been reported with varying frequency in observational studies of tuberculosis patients treated with clofazimine (Padayatchi et al., 2014; Dalcolmo et al., 2017; Trebucq et al., 2018; Misra et al., 2019), but causal inference is confounded by the co-administration of several other medications that can cause similar symptoms. The incidence of gastrointestinal symptoms in controlled trials did not differ significantly between those receiving and those not receiving clofazimine (Tang et al., 2015; Duan et al., 2019; Du et al., 2020). Overall, gastrointestinal symptoms appear to be mostly mild, with no reports of clofazimine being stopped due to gastrointestinal intolerance during DR-TB treatment.

Long-term studies in leprosy patients have not found evidence of clinically significant abnormalities in haematological, renal, hepatic or pancreatic blood parameters (Hastings et al., 1976; Costa Queiroz et al., 2002). In the four Chinese randomized trials among DR-TB patients (Tang et al., 2015; Wang et al., 2018; Duan et al., 2019; Du et al., 2020), the rate of haematological abnormalities, renal impairment and hepatic injury did not differ significantly between study arms, except in one trial where liver function test abnormalities were reported in 12% (8/66) of patients in the clofazimine group compared with 3% (2/74) in the standard of care group (*p* = 0.046) (Duan et al., 2019). Taken together, clofazimine does not appear to require routine laboratory investigations other than periodic monitoring of liver function tests.

### New developments

Much effort has been dedicated to developing clofazimine derivatives with improved pharmacokinetic and toxicity profiles by targeting less lipophilic compounds, anticipated to cause less tissue accumulation and discolouration as well as improved oral bioavailability (Jagannath et al., 1995; Reddy et al., 1996; van Rensburg et al., 2000; Kamal et al., 2005; Lu et al., 2011; Zhang et al., 2012b; Li et al., 2012; Liu et al., 2012; Zhang et al., 2014; Xu et al., 2019; Bvumbi et al., 2021; Saravanan et al., 2021). An attempt has also been made to alter the intrinsic colour, but this required modifications to the phenazine core, which eliminated antituberculosis activity (Liu et al., 2012). Of the hundreds of analogues synthesized and screened, several have been identified with equivalent or greater *in vitro* and animal model *in vivo* activity against *M. tuberculosis* than clofazimine while reportedly also producing less tissue discolouration and no other overt toxicity (Jagannath et al., 1995; Reddy et al., 1996; van Rensburg et al., 2000; Lu et al., 2011; Zhang et al., 2012b; Liu et al., 2012). One novel riminophenazine, TBI-166 (also called pyrifazimine), has advanced to clinical evaluation (ChiCTR 1800018780, 2018; NCT04670120, 2020). TBI-166 demonstrated activity equivalent to clofazimine in mice and produced less discolouration (Xu et al., 2019). Several TBI-166-containing combination regimens have been evaluated *in vitro* and mice demonstrating synergistic activity with the same companion drugs as clofazimine (Zhang et al., 2019; Ding et al., 2022). The combination of TBI-166 plus bedaquiline and pyrazinamide has been identified as the most potent TBI-166 combination evaluated, showing sterilizing activity comparable to the BPaL regimen in a mouse model (Ding et al., 2022). One study evaluated Rv0678 mutations as a mechanism of TBI-166 resistance and found these caused a lower fold change in MIC for TBI-166 than for both clofazimine and BDQ (Xu et al., 2019). Spontaneous resistance to TBI-166 was reported in *M. tuberculosis* wild-type strains, but the genetic basis for this has not been studied (Xu et al., 2019). Another novel analogue was recently described which maintained activity against a strain resistant to clofazimine, suggesting the possibility of a different mechanism of action (Zhao et al., 2022). In view of the riminophenazines’ unique mechanism of action and synergistic activity with the combination of bedaquiline and pyrazinamide, the prospect of a novel riminophenazine analogue producing less skin discolouration that has advanced to the clinical evaluation stage is exciting and ongoing efforts to achieve this goal remain important.

Novel drug delivery strategies are another approach being pursued to overcome some of the limiting properties of clofazimine. Clofazimine can be encapsulated in liposomes, allowing for parenteral administration, which is not possible with the free drug due to its low aqueous solubility (Mehta et al., 1993). In murine models, intravenously administered liposomal clofazimine increased the maximum tolerated dose by 8-fold (Mehta, 1996). Intravenous liposomal clofazimine at a dose of 50 mg/kg showed significantly better *in vivo* therapeutic activity against *M. tuberculosis* than the maximum tolerated dose of the free drug (Mehta, 1996; Adams et al., 1999). Other strategies to improve bioavailability or enable parenteral administration (both intravenous and inhaled) include nanocrystalline suspensions and nanoparticle encapsulation of clofazimine (Peters et al., 2000; Verma et al., 2013; Valetti et al., 2017; Murashov et al., 2018b; Chen et al., 2018). In one study, twice weekly inhaled clofazimine showed some efficacy against *M. tuberculosis* in a mouse model (Verma et al., 2013) and in another study, an intravenously administered formulation appeared to completely circumvent skin discolouration (Murashov et al., 2018b). Nanoparticle-based targeted delivery of clofazimine aimed at improving intra-macrophage activity (Pawde et al., 2020) and central nervous system penetration (de Castro et al., 2021) has also been described. TBM is one of the specific clinical scenarios where these optimised drug delivery strategies may theoretically be of value. However, none of these strategies has advanced beyond the early preclinical stage, likely due to their relative niche, albeit important, applications.

## Discussion

Clofazimine entered clinical use without the rigorous pharmacokinetic and safety evaluation which is part of modern drug development. It is hampered by extremely low aqueous solubility, leading to erratic absorption and low plasma concentrations. It has a very long elimination half-life and accumulates extensively in certain tissues leading to skin discolouration and drug crystal deposition in macrophages. However, due to its potent activity against *M. tuberculosis* strains resistant to rifampicin and isoniazid, clofazimine has become widely used in DR-TB treatment over the past decade. Despite its apparent lack of early bactericidal activity, clofazimine contributes synergistic sterilizing activity and treatment-shortening potential to several first- and second-line drug combination regimens. Clofazimine’s mechanism of action appears to be multi-modal and is likely related to its interaction with the mycobacterial respiratory chain leading to a combination of intracellular pro-oxidative effects, and disruption of cellular respiration and potassium uptake. Resistance to clofazimine still appears to be relatively uncommon, but is driven to some extent by cross-resistance with bedaquiline and is therefore likely to increase with increasing use of bedaquiline and clofazimine in TB programmes. For this reason, drug susceptibility testing is necessary for patients with prior exposure to these drugs, and population-level surveillance should be undertaken in high-burden settings where these drugs are used programmatically to monitor the emergence of population-level resistance to these key drugs. Clofazimine-induced skin discolouration is the most frequent adverse effect of the drug, and though it is regarded as a cosmetic rather than a safety concern, it can potentially lead to stigma and may have a profound impact on psychological wellbeing and potentially pose a risk to treatment adherence. The advancement of pyrifazimine, a less lipophilic clofazimine analogue reportedly causing less skin discolouration, into early-phase clinical testing is an encouraging prospect toward improving the tolerability of riminophenazines. The QT prolonging of clofazimine, causing QT prolongation, especially when combined with other QT prolonging drugs such as bedaquiline and fluoroquinolones, areimportant, but infrequently result in clinically significant events (Hewison et al., 2022) and need to be weighed up against the risks associated with alternative drug choices. Electrocardiographic monitoring is indicated when clofazimine is combined with other QT-prolonging drugs. Despite the body of evidence supporting its safety and efficacy for DR-TB treatment and over a decade of used in many national programmes, clofazimine is not yet registered for tuberculosis treatment in several countries, still requiring off-label use and creating a barrier to access in these jurisdictions.

As the incidence of drug-resistant tuberculosis rises and resistance to new and repurposed drugs emerges, the value of each antituberculosis drug class with a distinct mechanism of action cannot be underestimated. For this reason, despite the limitations of clofazimine, the riminophenazines remain important, whether for individualized therapy in people with difficult-to-treat DR-TB or potentially as a first-line agent as part of a novel, shorter DS-TB or DR-TB regimens. In this context, the development of novel riminophenazine analogues with equivalent activity but an improved pharmacokinetic and tolerability profile to eventually replace clofazimine will be highly desirable and efforts toward their discovery and development for clinical use should be a priority.
